# Ankle-Foot Orthosis Made by 3D Printing Technique and Automated Design Software

**DOI:** 10.1155/2017/9610468

**Published:** 2017-07-30

**Authors:** Yong Ho Cha, Keun Ho Lee, Hong Jong Ryu, Il Won Joo, Anna Seo, Dong-Hyeon Kim, Sang Jun Kim

**Affiliations:** ^1^Department of Physical Therapy, Samsung Medical Center, Seoul, Republic of Korea; ^2^Department of Occupational Therapy, Samsung Medical Center, Seoul, Republic of Korea; ^3^Research Planning Team, SolidEng Corporation, Incheon, Republic of Korea; ^4^3D Convergence Technology Center, Kyungpook National University, Daegu, Republic of Korea; ^5^Department of Physical and Rehabilitation Medicine, Sungkyunkwan University School of Medicine and Samsung Medical Center, Seoul, Republic of Korea

## Abstract

We described 3D printing technique and automated design software and clinical results after the application of this AFO to a patient with a foot drop. After acquiring a 3D modelling file of a patient's lower leg with peroneal neuropathy by a 3D scanner, we loaded this file on the automated orthosis software and created the “STL” file. The designed AFO was printed using a fused filament fabrication type 3D printer, and a mechanical stress test was performed. The patient alternated between the 3D-printed and conventional AFOs for 2 months. There was no crack or damage, and the shape and stiffness of the AFO did not change after the durability test. The gait speed increased after wearing the conventional AFO (56.5 cm/sec) and 3D-printed AFO (56.5 cm/sec) compared to that without an AFO (42.2 cm/sec). The patient was more satisfied with the 3D-printed AFO than the conventional AFO in terms of the weight and ease of use. The 3D-printed AFO exhibited similar functionality as the conventional AFO and considerably satisfied the patient in terms of the weight and ease of use. We suggest the possibility of the individualized AFO with 3D printing techniques and automated design software.

## 1. Introduction

An ankle-foot orthosis (AFO) is widely used for foot drop caused by peroneal neuropathy. Conventional manufacturing of AFO consists of manual plaster casting, molding of thermoplastic materials, and cutting them as a form of AFO, which requires delicate skill and much effort. In addition, the whole process of this manufacturing must be repeated if the AFO is destroyed or a patient's condition is changed.

Three-dimensional (3D) printing technique, also known as additive manufacturing, has been widely used in medical fields, and their use is growing explosively. Three-dimensional printers can produce easily modifiable objects without any fixed moldings, which make the objects unique. Artificial organs using bioprinters [1, 2], titanium instrumentation for skull defect [3] and hip arthroplasty [4, 5], and hand prosthesis for amputation [6, 7] are examples used in the medical fields. This 3D printing technique makes it possible for physicians and surgeons to create easily patient-tailored products for themselves [8].

Recently, several trials to manufacture an AFO using 3D printing technique have been done [9–11]. An orthosis made with 3D printing techniques has advantages in less delicate skill and effort to manufacture and easy reproduction over conventionally manufactured orthosis made by molding the thermoplastic material [12]. Moreover, as the designed 3D modelling file is stored once, manufacturing of an AFO can be easily repeated. In addition, if an automated software program for the design of orthosis is developed using the preprogrammed orthotic template design, the production of the orthosis will be easily achieved and can be modified by patients for themselves.

We developed an automated software program for the design of orthosis and manufactured the orthosis using the 3D printing technique. In this study, we described this manufacturing process and clinical results after the application of this AFO to a patient with a foot drop.

## 2. Materials and Methods

### 2.1. Patient

A 68-year-old woman visited our department for foot drop on the right side after an embolectomy in July 2015. The cause of the foot drop was diagnosed by an electrodiagnostic study as common peroneal neuropathy, with a complete axonotmesis state at the fibular head level. A physical examination revealed that the motor power of the ankle dorsiflexion was nil, and the gait pattern was a steppage gait with the assistance of a single cane. She already had a conventional AFO for foot drop but rarely used it because it was heavy and uncomfortable both outdoors and indoors. She agreed to participate in our 3D printing study.

### 2.2. Procedures

To manufacture an AFO using 3D printing technique, scanning of the patient's lower right leg was done with a 3D scanner, Eva (Artec™ Eva, Artec Group, Luxembourg), in order to design the AFO. Before the scanning process, the patient was asked to stand with her foot in a neutral position in the sagittal and coronal planes because the longitudinal, transverse, and metatarsal arches must be considered when designing the AFO. In the standing position, the scanning procedure was performed with a 3D scanner, and in the sitting position, the scanning procedure was repeated for the masked areas of the lower leg not shown in the standing position. The scanner captures up to 16 frames per second, and these frames are aligned automatically in real-time, which makes scanning easy and fast.

**Figure 1:**
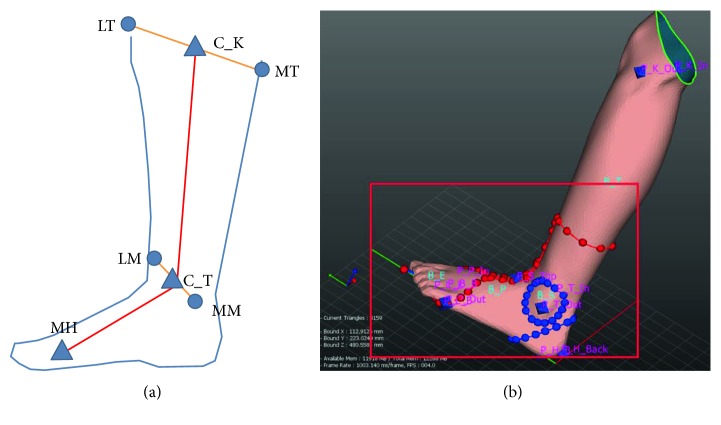
The line between the second metatarsal head (MH) and the midpoint (C_T) of the lateral (LM) and medial malleoli (MM) was assumed to be the axis of the foot, and the line between the midpoint (C_T) of the lateral (LM) and medial malleoli (MM) and the midpoint (C_K) of the lateral (LT) and medial tibial condyles (MT) was assumed to be the axis of the lower leg (a). The preprogrammed orthotic template design which size was modified according to the marked points was overlapped (red dots and lines), the circles were drawn around the lateral and medial malleoli, and the oblique line was drawn (blue dots and lines (b)).

**Figure 2:**
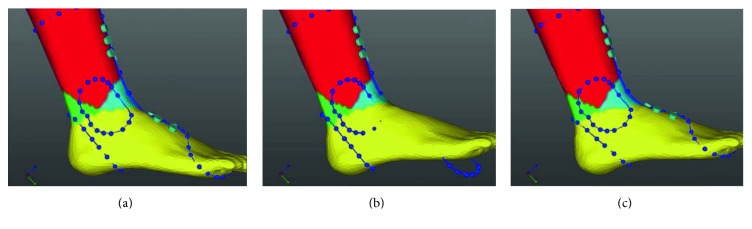
In the sagittal view, based on the axes of the foot and lower leg, the ankle joint was adjusted to a neutral position by dorsiflexion (b) and the templates were adjusted according to the meshes (c).

**Figure 3:**
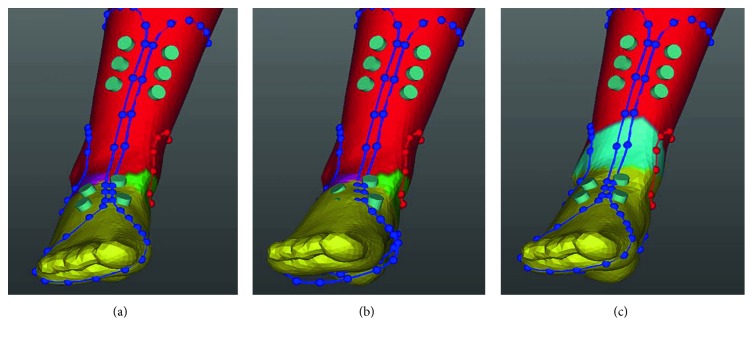
In the coronal view, based on the axes of the foot and lower leg, the ankle joint was adjusted to a neutral position by eversion (b) and the templates were adjusted according to the meshes (c).

**Figure 4:**
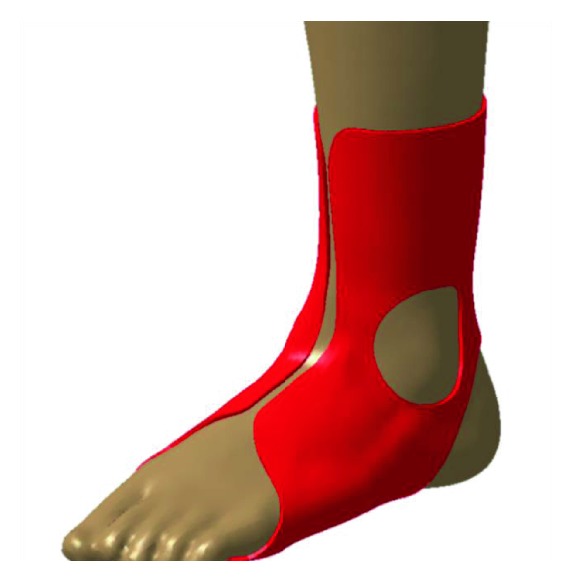
Using the adjustment of the joint axis, the 3D modelling of individualized AFO with correction of the axis was designed.

After acquiring a 3D modelling file, orthosis software (MediACE3D®, SolidEng Corp., Daejeon, Korea) loaded this modelling file and created the “STL” file for preparation of the customized AFO. This automated software program was based on the anthropometric data of normal, healthy volunteers and the preprogrammed orthotic template design. The points for the anatomic landmark were manually marked at the heel, the first and the fifth metatarsal heads, the second metatarsal head, lateral and medial malleoli, and lateral and medial tibial condyles. The line connecting the second metatarsal head to the midpoint between the lateral and medial malleoli was determined to be the axis of the foot, and the line connecting the midpoint between the lateral and medial malleoli to the midpoint between the lateral and medial tibial condyles was determined to be the axis of the lower leg (Figure 1(a)). The preprogrammed orthotic template design which size was modified according to the marked points including the first and the fifth metatarsal heads was overlapped, and the circles of which radius was predetermined were drawn around the lateral and medial malleoli, and the oblique line of which angle was 45 and at the predetermined distance separates from the heel was drawn (Figure 1(b)). Based on the axes, the ankle joint was adjusted to a neutral position by dorsiflexion (Figure 2(b)) and the templates were adjusted according to the meshes (Figure 2(c)). Ankle joint and templates were also adjusted to a neutral position by eversion (Figure 3()). Using this algorithm, the 3D modelling of individualized AFO was designed (Figure 4()). The process of creating the “STL” file using the automated software program was presented in the dynamic file (supplementary file available online at https://doi.org/10.1155/2017/9610468).

The designed AFO was printed using a fused filament fabrication (FFF) type 3D printer (FB9600®, TPC Mechatronics Corp., Incheon, Korea). The FFF method accumulates the thermoplastic filaments layer-by-layer as the filaments melts. In this study, one of the thermoplastic filaments, thermoplastic polyurethane (Shenzhen Esun Industrial Co. Ltd, Shenzhen, China), which was nontoxic and highly flexible, was used (diameter 1.75 mm, extruders temperature 210–230°C).

**Figure 5:**
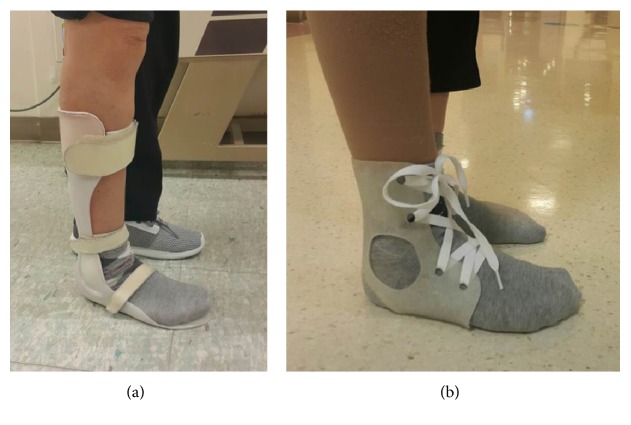
The ankle-foot orthosis made by automated software program was applied to the patient. Shoe laces were used to wear the 3D-printed AFO (b). A conventional AFO without a joint that was made from polypropylene was used for the control (a).

After printing out the designed AFO, postprinting process was done to remove the support structures and to smoothen the surface by the pincer and sandpaper. This postprinting process was performed by an experienced orthotist. After the postprinting process, the AFO orthosis was delivered and applied to the patient. Shoe laces were used to wear the 3D-printed AFO (Figure 5). A conventional AFO without a joint that was made from polypropylene was used for the control (Figure 5).

### 2.3. Evaluation

**Figure 6:**
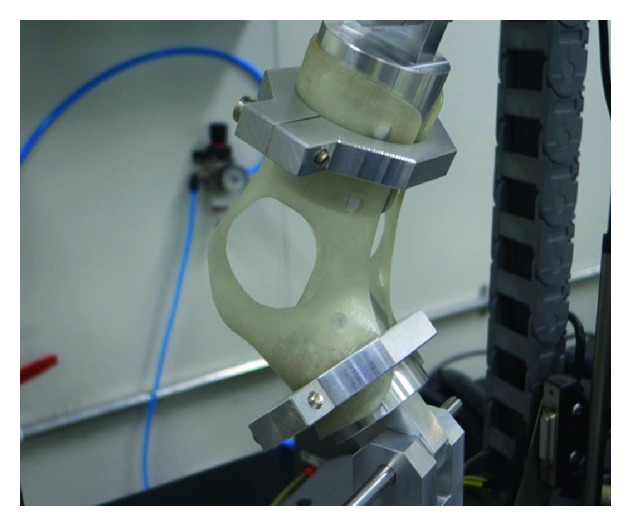
To evaluate the durability of the 3D-printed AFO, a mechanical stress test was performed. At both ends of the AFO orthosis made for the test, round-shaped plastic dummies were inserted and affixed to the machine.

To evaluate the durability of the 3D-printed AFO, a mechanical stress test was performed. At both ends of the AFO orthosis made for the test, round-shaped plastic dummies were inserted and affixed to the machine (Figure 6). The stress ratio was 0.1, stretching force was set to 50 N, the frequency was 1 Hz with sine waves, and the total repetition time was 300,000 cycles. Stretching force was selected to simulate the partial body weight to endure the stance phase, and 1 Hz frequency was selected to simulate the cadence of walking. A total of 300,000 cycles were selected to simulate the use of AFO by a patient. If a patient walks 2500 steps every day with AFO, 300,000 cycles represent 4 months of activity.

For the comparison, the patient alternated between the 3D-printed AFO and conventional AFO for two months. After two months, the patient visited the outpatient clinic and was tested using the Quebec User Evaluation of Satisfaction with Assistive Technology (QUEST) [13].

To evaluate the clinical usefulness of the 3D-printed AFO, the kinematic and dynamic electromyographic analyses were evaluated using a 3D gait analysis (HWK-200RT®, Motion Analysis Corp., USA). The gait analysis was performed with the 3D-printed AFO, with the conventional AFO, and without the AFO; all the situations were repeated three times to enhance the test reliability.

This study was approved by our institutional review board (Approval no. 2014–12-013), and informed consent was acquired from the patient.

## 3. Results

There was no crack or damage after 300,000 repetitions in the durability test. After the durability test, the shape and stiffness of the AFO did not change.

The gait analysis showed that the gait speed increased after wearing the conventional AFO (56.5 cm/sec) and 3D-printed AFO (56.5 cm/sec) compared to that without an AFO (42.2 cm/sec). The stride length also increased after wearing the conventional AFO (70.9 cm) and 3D-printed AFO (70.9 cm) compared to that without an AFO (63.2 cm). The step width decreased when the patient walked with the 3D-printed AFO (15.9 cm) compared to the step width with the conventional AFO (17.1 cm) and without an AFO (17.9 cm). The single stance ratio between the left and right sides was most symmetric for the 3D-printed AFO (80.4%), followed by the conventional AFO (79.7%), and it was least symmetric without an AFO (69.7%).

**Figure 7:**
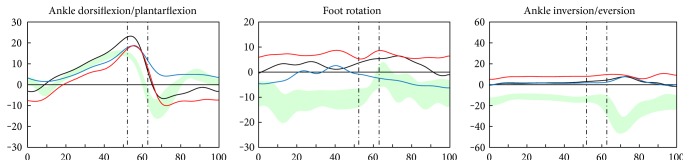
The conventional AFO (blue line) caused the ankle to be in a more dorsiflexed state in the swing phase, compared to the 3D-printed AFO (black line) and without AFO (red line), which caused the least dorsiflexed state. The foot rotation in the transverse plane was corrected the most with the conventional AFO, followed by the 3D-printed AFO, and it was least corrected without an AFO. The ankle eversion was corrected the same with the conventional and 3D-printed AFO.

The kinematic analysis showed that the conventional AFO caused the ankle to be in a more dorsiflexed state in the swing phase, compared to the 3D-printed AFO and without AFO, which caused the least dorsiflexed state (Figure 7). The foot rotation was corrected the most with the conventional AFO, followed by the 3D-printed AFO, and it was least corrected without an AFO (Figure 7). The ankle eversion was corrected the same with the conventional and 3D-printed AFOs (Figure 7).

According to the QUEST, all of the items were ranked as “very satisfied” or “satisfied.” The patient was more satisfied with the 3D-printed AFO than the conventional AFO in terms of the weight and ease of use, while the conventional AFO was more effective than the 3D-printed AFO because the material of the 3D-printed AFO was flexible. Specific comments are described in Table 1.

## 4. Discussion

In our study, we designed and manufactured an AFO with 3D printing techniques and using the automated design CAD software. This 3D-printed AFO exhibited similar functionality as the conventional AFO and considerably satisfied the patient in terms of the dimension, weight, adjustment, ease of use, and comfort.

There was no crack or damage after 300,000 repetitions in the durability test. We set 300,000 repetitions (4-month duration) although these are not enough to test the durability of the orthosis because of the test time, cost, and availability of the test machine. In addition, stress–strain curve was not drawn because we focused on the crack or damage in this study. We investigated the change of shape visually and did not find any change during the application before and after the durability test. Later studies for more repetitions and stress-strain curve will be necessary to investigate the accurate rheological properties of an AFO.

In our results, the gait speed, cadence, and stride length improved following application of the 3D-printed AFO compared to without AFO being used and this improvement was similar to the conventional AFO. Creylman et al. [14] also made a 3D-printed AFO by using a selective laser sintering technique and demonstrated that this had the same function as a conventional AFO made from polypropylene and was superior to the conventional AFO. These results were similar to our results in terms of the function and comfort.

Our results demonstrated that the single stance ratio between the left and right sides was symmetric in the 3D-printed AFO compared to the conventional AFO and without AFO. This makes the patient walk more naturally and with more stability and implies that the 3D-printed AFO was more functional.

The 3D-printed AFO caused ankle dorsiflexion and prevented foot drop during the swing phase but not as much as the conventional AFO did. This suggests that the 3D-printed AFO was inferior to the conventional AFO for preventing foot drop. The patient also expressed less effectiveness with the 3D-printed AFO than the conventional AFO. This was due to the decreased traction force that resulted from the thermoplastic elastomer stretching. Therefore, we believe that the 3D-printed AFO must be designed in a more dorsiflexed position to compensate for this stretching.

Our 3D-printed AFO focused on the weight, individualization, and comfort rather than the function. Therefore, all of the items in the QUEST showed better results than the conventional AFO. In addition, our 3D-printed AFO had the advantage of being easily wearable inside a shoe compared to the conventional AFO, which usually requires larger shoes to wear.

The automated orthosis software used in this study suggests the possibility for medical facilities to design and print individualized AFOs with a one-step process. To make this possible, the postprinting process must be solved because removal of the support materials and smoothing of the surface require much skill and effort. If the postprinting process becomes simple and easy, then a one-step process for manufacturing the orthosis will be accomplished in the future.

This was a case study, so we could not use the statistical methods. To solidify our conclusion, a randomized controlled or cross-over study must be performed. However, we suggest the possibility of the individualized AFO with 3D scanning and printing techniques and this will become popular in the future.

## Supplementary Material

The supplementary dynamic file shows the whole process from the selection of a patient to the production of the programmed “STL” file. The processes were 1) selection of a patient, 2) determination of the points, 3) loading the preprogrammed template, 4) adjustment of the axes, and 5) production of the programmed “STL” file.

## Figures and Tables

**Table 1:** Quebec User Evaluation of Satisfaction with Assistive Technology (QUEST) after application of 3D-printed and conventional ankle-foot orthosis.

Item	3D-printed AFO		Conventional AFO	
	Score	Comment	Score	Comment
(1) Dimensions	4	Need to support the anterior part of the foot	4	Difficult to wear due to size
(2) Weight	5		3	Heavy to wear
(3) Adjustment	4	Slight difficult to tie shoe laces	5	
(4) Safety	5		5	
(5) Durability	5		5	
(6) Easy to use	5		2	Difficult to wear due to thickness
(7) Comfort	4	Mild unstable feeling	4	Uncomfortable due to hardness
(8) Effectiveness	4	Mild foot drop during the swing phase	5	
Total satisfaction	4.5		4.1	
Most important 3 items		(1) Weight, (2) dimension, and (3) safety		
